# Aspen impedes wildfire spread in southwestern United States landscapes

**DOI:** 10.1002/eap.70061

**Published:** 2025-07-07

**Authors:** Matthew P. Harris, Jonathan D. Coop, Jared A. Balik, Jessika R. McFarland, Sean A. Parks, Camille S. Stevens‐Rumann

**Affiliations:** ^1^ Clark School of Environment and Sustainability Western Colorado University Gunnison Colorado USA; ^2^ Aldo Leopold Wilderness Research Institute Rocky Mountain Research Station, USDA Forest Service Missoula Montana USA; ^3^ W.A. Franke College of Forestry and Conservation University of Montana Missoula Montana USA; ^4^ Forest and Rangeland Stewardship and Colorado Forest Restoration Institute Colorado State University Fort Collins Colorado USA

**Keywords:** broadleaf forest, climate change, conifer forest, fuel treatment, green fuel break, quaking aspen, resilience, stabilizing feedback, wildfire

## Abstract

Aspen (*Populus tremuloides*) forests are generally thought to impede fire spread, yet the extent of this effect is not well quantified in relation to other vegetation types. We examined the influence of aspen cover on interpolated daily fire spread rates, the relative abundance of aspen at fire perimeters versus burn interiors, and whether these relationships shifted under more fire‐conducive atmospheric conditions. Our study incorporated 314 fires occurring between 2001 and 2020 in the southwestern United States and a suite of gridded vegetation, topography, and fire weather predictor variables. We found that aspen slows fire progression: as aspen cover on the landscape increased, daily area burned and linear spread rate decreased. Where aspen cover was <10%, daily fire growth averaged 1112 ha/day and maximum linear spread was 2.1 km/day; where aspen exceeded 25%, these values dropped to 368 ha/day and 1.3 km/day. Aspen also serves as a barrier to fire spread, demonstrated through a higher proportion of aspen cover at fire perimeters than in burn interiors. Finally, though favorable fire weather conditions increased fire growth rates, differences between aspens and conifers persisted. Our results affirm that aspen stands can act as a firebreak, with clear applications for vegetation management. For example, interventions that shift conifer to aspen cover could lessen the risk of fire for nearby values at risk (e.g., communities, infrastructure) but still support forest ecosystem function. Further, wildfire‐driven conversion from conifer to aspen forest types in some landscapes may produce a negative feedback that could dampen expected increases in fire activity under a warmer and drier climate.

## INTRODUCTION

Fire–vegetation feedbacks can stabilize or disrupt ecosystems, yet our understanding of these processes and how they respond to human influences and climate change is limited. Fire is a keystone natural process that shapes vegetation composition, structure, and spatial pattern; in turn, vegetation exerts a fundamental control over fire likelihood, behavior, and effects (Bowman et al., 2020; Loudermilk et al., 2022). For example, frequent surface fires can maintain grassland and savanna‐like vegetation that promotes frequent burning (Beckage et al., 2009). Similarly, infrequent but severe fires can reduce fuels and drive shifts in woody vegetation types that inhibit near‐future burning (Cansler et al., 2022), elongating fire return intervals but ultimately supporting severe future fire. However, human ignitions and fire suppression practices can shift these balances (Kreider et al., 2024), producing both fire surpluses and deficits (Donovan & Brown, 2007; Parisien et al., 2020; Steel et al., 2015), with major implications for fuels, vegetation, and subsequent fire activity. Recent warming and drying are further expanding wildfire activity in many regions (Parks & Abatzoglou, 2020; Senande‐Rivera et al., 2022), with the potential to drive rapid and extensive vegetation change with subsequent feedbacks on future fire regimes (Hurteau et al., 2019; Johnstone et al., 2016). Accordingly, an improved understanding of the extent to which vegetation can modulate fire activity, particularly under increasingly fire‐conducive weather and climate conditions, can inform anticipated future changes and leverage points for management interventions (Harris et al., 2016; Hessburg et al., 2021).

In western North America, increasing fire activity and changing post‐fire climate are poised to drive changes to a wide range of ecological systems with implications for future fire activity. Recent increases in annual area burned are attributed to warmer and drier fire seasons, and projections suggest even more growth in fire activity as climate warming continues (Abatzoglou & Williams, 2016; Coop et al., 2022). Increasing area burned at high severity (Parks & Abatzoglou, 2020; Singleton et al., 2019) and declining post‐fire conifer regeneration under warmer and drier post‐fire climate (Davis et al., 2023) may set the stage for extensive conversion of conifer forests to alternate forest types or non‐forest vegetation (Coop et al., 2020; Guiterman et al., 2022). In some settings (e.g., southwestern US montane forests), conifers may be replaced by broadleaf forest types such as aspen (*Populus tremuloides* Michx.) that could impede future burning due to reduced flammability in comparison with conifers (Nesbit et al., 2023).

Relative to conifer forests, aspen stands are thought to be resistant to burning due to higher moisture content in their foliage and understory, branches held high above the understory, and chemical differences that reduce flammability (Nesbit et al., 2023). By contrast, conifer forests are generally more fire‐prone due to abundant fine fuels, flammable resins, and more continuous horizontal and vertical fuel structure (Nesbit et al., 2023; Popović et al., 2021; Varner et al., 2022). However, relative differences in flammability between aspen and conifer forests may vary with landscape factors and climatic influences. Young aspen stands are likely more fire resistant due to a greater proportion of live standing biomass, and older stands are more flammable due to accumulating dead and downed biomass and conifer encroachment (Alexander & Sando, 1989; Rogers et al., 2014; Shinneman et al., 2013). Further, fire weather may modulate differences between aspen and conifer forest flammability, though this has not been empirically assessed. In a simulation study, DeRose and Leffler (2014) found that differences between aspen and conifer forest fire behavior were pronounced under moderate fire weather but diminished under extreme weather that produced crown fires in both types. Strengthening atmospheric vapor pressure deficit and increasingly extreme fire weather under warmer and drier climate may override fire–vegetation feedbacks and reduce the influence of forest type on fire progression (Abatzoglou et al., 2021; Cawson et al., 2024).

Increasing risks posed to communities and ecosystems from expanding wildfire activity have compelled the development and implementation of a suite of vegetation management interventions. Fuel reduction treatments increase the height to live crowns, decrease tree density, and limit fuel continuity, which reduces torching and running crown fires (Agee & Skinner, 2005). In the western United States, shaded fuel breaks produced by mechanical thinning are the most common treatment, though fuel breaks using a combination of thinning and prescribed fire are the most effective at moderating fire activity (Davis et al., 2024; Fernandes, 2015; Urza et al., 2023). As an alternative to shaded fuel breaks, green fuel breaks incorporating forest cover of low‐flammability tree species may also limit the spread of crown fires (Wang et al., 2021). Firebreaks composed of aspen within a matrix of conifer forest are expected to offer an approach to limiting fire spread and protecting human communities (Brown, 1989; Cui et al., 2019; Curran et al., 2017; Fechner & Barrows, 1976). However, the extent to which aspen may slow or stop fire spread relative to conifer forest types has not been well quantified (Nesbit et al., 2023).

The widespread availability of remotely sensed data on fire activity, combined with detailed forest cover maps and gridded weather data, offers new opportunities to empirically assess how vegetation influences fire behavior at landscape scales (Chuvieco et al., 2020; Szpakowski & Jensen, 2019). The purpose of this study is to quantify the influence of aspen on fire progression to answer two questions: (1) What is the effect of aspen cover on fire spread rates, as measured from day‐of‐burning maps interpolated from satellite fire detections? (2) How effective is aspen at stopping fire entirely, as inferred by the relative abundance of aspen at fire perimeter boundaries versus burn interiors? We further examine how these relationships may be influenced by variation in fire weather, drought, and topography. Better understanding the influence of aspen cover on fire growth and extent will help inform future predictions of fire activity as wildfire drives shifts in vegetation (e.g., to what extent may shifts from conifer to aspen forests produce expected negative feedbacks on future fire?), and this knowledge can also guide management interventions such as green fuel breaks that could reduce undesired effects of wildfire to forest ecosystems and human communities.

## METHODS

### Study area

**FIGURE 1:**
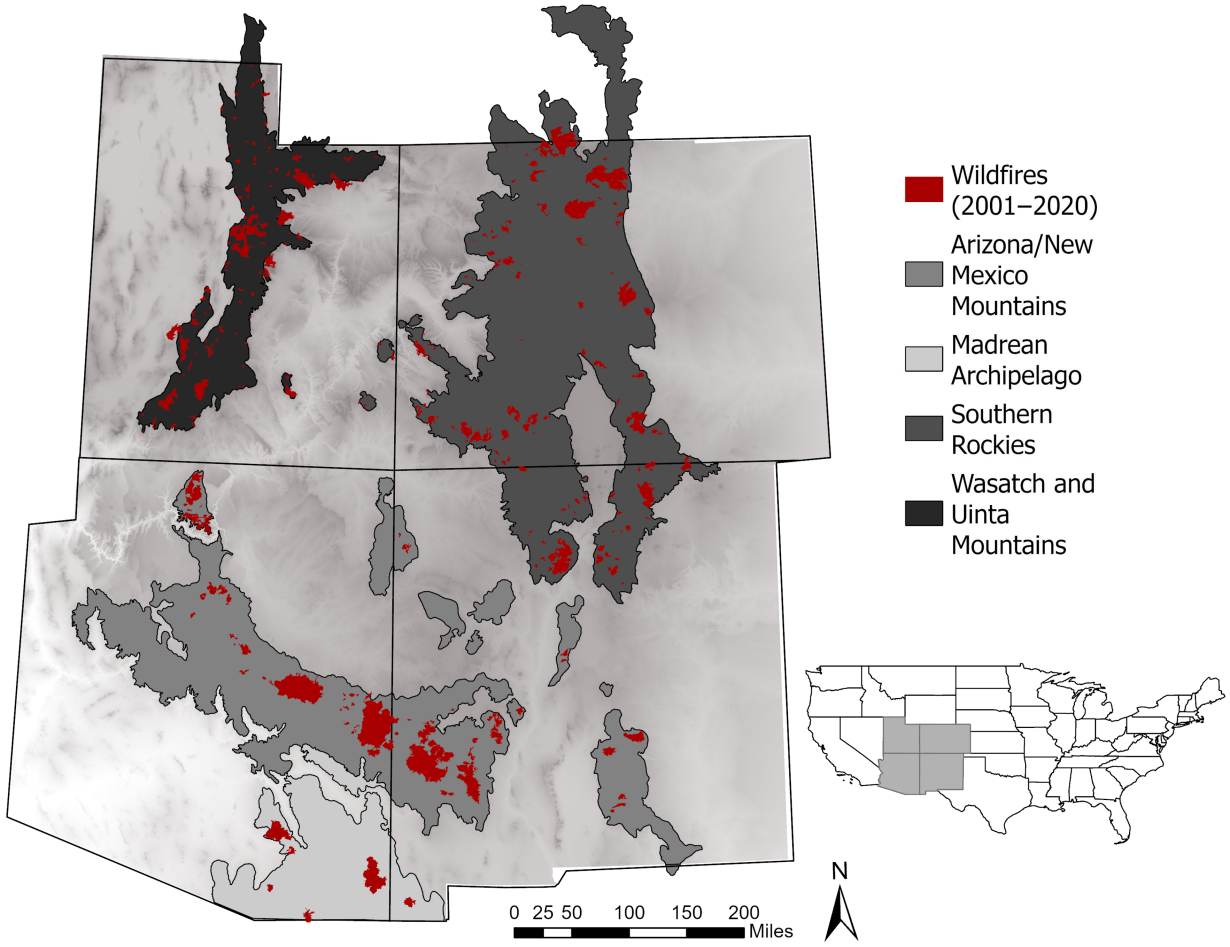
Study area in the southwestern United States, showing forested EPA Level III Ecoregions and sampled fires occurring between 2001 and 2020.

Our study area encompasses forested areas of the Four Corners region (Arizona, Colorado, New Mexico, and Utah) of the southwestern United States (Figure 1). In particular, our analysis includes the following Environmental Protection Agency Level III Ecoregions: the Arizona/New Mexico Mountains, Madrean Archipelago, Southern Rockies, and Wasatch and Uinta Mountains (U.S. Environmental Protection Agency, 2013). This semiarid region is characterized by steep climate gradients associated with elevational and topographic variation (Barton, 1994), which in turn drive variation in vegetation. Desert lowlands, arid grasslands, and sagebrush steppe give way at progressively higher elevations to a broad range of forest types including piñon–juniper (*Pinus* and *Juniperus* spp.) woodlands, warm conifer forests dominated by ponderosa pine (*Pinus ponderosa*) and Douglas‐fir (*Pseudotsuga menziesii*), montane forests of aspen and lodgepole pine (*Pinus contorta*), and subalpine spruce–fir (*Picea* and *Abies* spp.) forests at the highest elevations. These forest types were historically characterized by distinct fire regimes, with lower elevation warm and dry forests (e.g., ponderosa pine) generally experiencing frequent but low‐severity fires, intermediate‐elevation forest types experiencing a mixed‐severity fire regime, and high‐elevation forests (e.g., spruce–fir) burning infrequently but severely (Heyerdahl et al., 2001; Odion et al., 2014; Sherriff et al., 2001; Swetnam & Baisan, 1996). Temporally, precipitation patterns are strongly influenced by climate teleconnections, most prominently the El Niño Southern Oscillation (ENSO), which can exert strong controls on the likelihood and extent of fire (Swetnam, 1990).

In addition to climatic influences, historical and contemporary fire regimes in the southwestern United States have been strongly influenced by human inhabitants. This study area represents the traditional homelands for a wide range of Indigenous groups, many of whom used fire to varying extents to shape landscapes. Dendrochronological records indicate Indigenous fire management often defined local fire regimes and weakened fire–climate relationships at landscape scales (Roos et al., 2021, 2022). Fire regimes changed dramatically following Euro‐American settlement due to the decline of Indigenous ignitions (Liebmann et al., 2016; Taylor et al., 2016), extensive livestock grazing that removed surface fuels, and eventual modern fire suppression, resulting in increased fuel loading and homogenized forest landscapes (Donovan & Brown, 2007; Savage & Swetnam, 1990; Steel et al., 2015). Recent, rapid expansion in fire activity is largely associated with warming and drying conditions that decrease fuel moisture and increase fire season length, and in these fuel‐accumulated landscapes, these conditions contribute to more severe burning and extreme fire events (Abatzoglou & Williams, 2016; Cawson et al., 2024; Kreider et al., 2024; Parks et al., 2023).

### Spread analysis: Does aspen slow fire growth?

**FIGURE 2:**
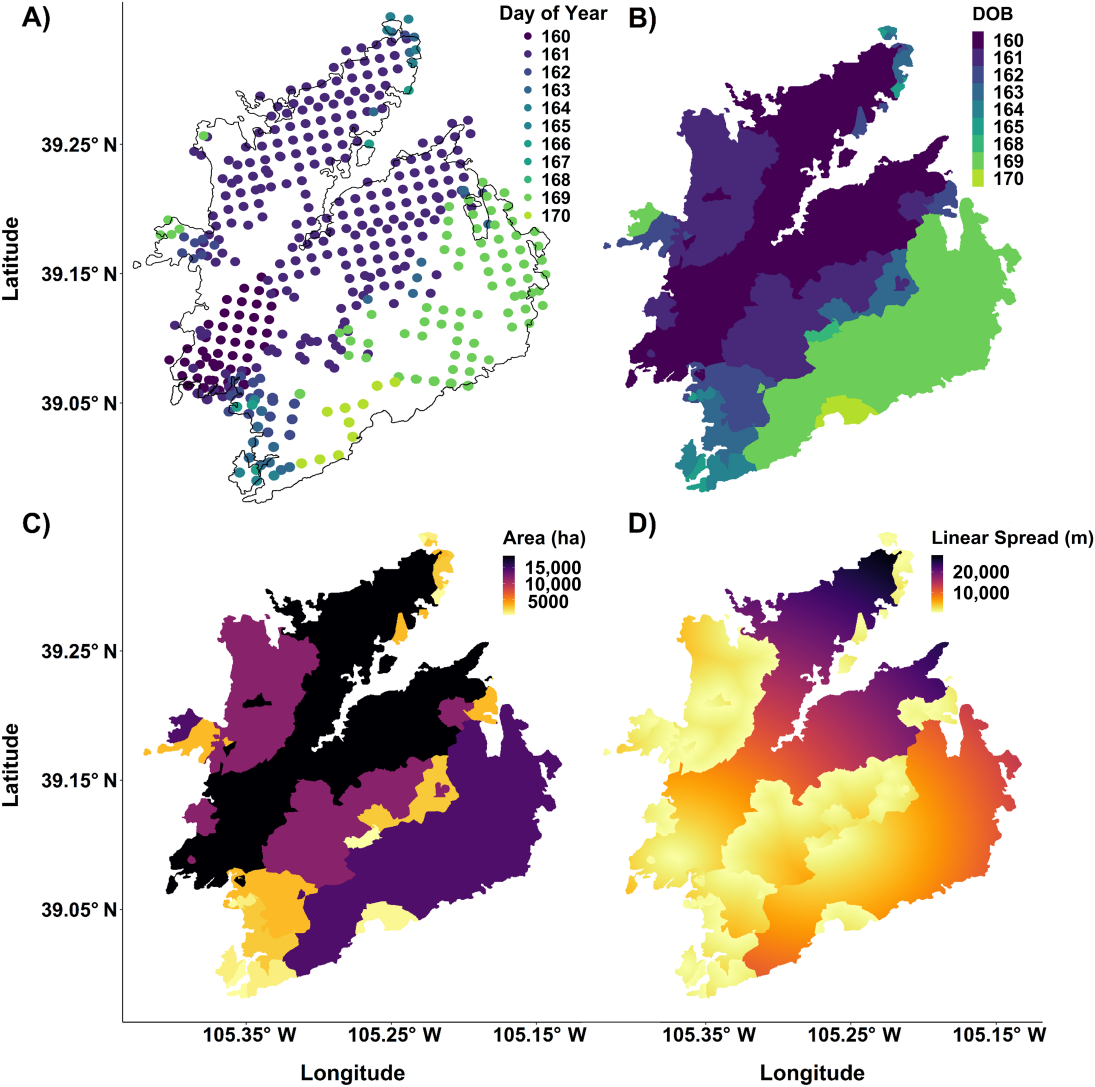
Illustration of how daily fire spread metrics were calculated, using the 2002 Hayman Fire in Colorado as an example: (A) final fire perimeter and daily MODIS fire detections, (B) day‐of‐burning (DOB) interpolations from MODIS fire detections, (C) area burned in each DOB (in hectares), and (D) rasterized linear spread (in meters); the maximum daily value was then assigned to all pixels within that DOB patch.

To address our first question of how aspen cover affects fire spread rate, we developed day‐of‐burning (DOB) interpolations based on the methods of Parks (2014). DOB interpolations are produced using Moderate Resolution Imaging Spectroradiometer (MODIS) and Visible Infrared Imaging Radiometer Suite (VIIRS) fire detections (from https://firms.modaps.eosdis.nasa.gov/active_fire/) to map daily fire progression at a 30‐m resolution (Figure 2A,B) within the final fire perimeter, acquired from Monitoring Trends in Burn Severity (MTBS; Picotte et al., 2020). The addition of VIIRS (beginning in 2012) to fire progression modeling follows several previous studies (Balik et al., 2024; Barber et al., 2024; Coop et al., 2022), and results in improved accuracy but overall has a negligible impact on our findings. We developed DOB interpolations for 205 unique fires that included at least 10 fire detections between 2002 and 2020 and included at least 0.1% of the aspen forest type (described below), producing 1687 spatially discrete DOB patches. The spread analysis has 109 fewer fires than the perimeter analysis (described below) as it begins a year later based on the first year of MODIS detections (2002 vs. 2001) and due to the minimum requirement for 10 fire detections. The DOB patches were used to produce two metrics of spread for each fire including (1) total daily area burned (in hectares) and (2) daily maximum linear spread (in meters). Daily area burned for each fire is defined as the total area within all DOB patches corresponding to a single 24‐h period as interpolated from MODIS and VIIRS fire detections (Figure 2C). Daily linear spread for any given day is measured as the rasterized distance (in meters) from the shared patch boundary of the previous day to the closest non‐shared patch boundary (forming the extent of that day's fire progression; Figure 2D). For the first day of a fire or any discontinuous day (lacking a shared boundary with a previous day, e.g., initiated by a spot fire), distance is measured from the centroid of that day's earliest MODIS or VIIRS fire detection to that day's boundary. Maximum daily linear spread is simply the greatest distance measured in a raster grid cell in each DOB patch.

Fire progression was related to the 30‐m resolution LANDFIRE Existing Vegetation Type (EVT) data (https://www.landfire.gov/evt.php; LANDFIRE, 2020a, 2022). Five versions of the EVT dataset spanned our study period: 2001, 2012, 2014, 2016, and 2020; we used the EVT version that was the most recent preceding each fire year. We reclassified 77 EVTs representing fine‐scale classifications into 10 coarse groups: *aspen, cool conifer* (e.g., spruce–fir forest types), *disturbed*, *herbaceous* (including alpine tundra), *mixed aspen–conifer*, *nonflammable* (e.g., bare rock), *shrub*, *warm conifer* (e.g., ponderosa pine and dry mixed‐conifer), *wetland*, and *woodland* (mostly pinyon–juniper) (Appendix S1: Table S1). The *warm conifer* group was the most prevalent (36.4%) with other groups such as *disturbed* (1.9%), *nonfuel* (1.2%), and *wetland* (1.8%) only accounting for a small portion of the analyzed area (Appendix S1: Table S2). Although the area accounted for by the aspen type was relatively stable across different LANDFIRE versions, we noted that the mixed aspen–conifer type (often representing stands undergoing succession from aspen to conifer types; NatureServe, 2018) changed substantially in the 2016 release. For this reason, we isolated mixed aspen–conifer as a separate group, with the primary focus of our study being the “pure” aspen cover type. To account for the influence that recent prior burns have on subsequent fire likelihood and spread (Holsinger et al., 2016), for each year of our analysis, we used previous MTBS burn perimeters to reclassify any area burned within the past 1–10 years as *prior burn*. The most recent LANDFIRE EVT preceding the fire was used to calculate the percent of each cover type, including aspen forest, occurring within each DOB patch.

To evaluate daily weather conditions as covariates influencing fire spread rates, we sampled the ERA5 reanalysis dataset (Hersbach et al., 2020). Daily variables included accumulated precipitation (AP), build up index (BUI), daily severity rating (DSR), drought code (DC), duff moisture code (DMC), fine fuel moisture code (FFM), fire weather index (FWI), initial spread index (ISI), max temperature (Tmax), relative humidity (RH), and wind speed (WS), all at a 31‐km resolution. Similarly, we sampled monthly climate variables including climatic water deficit (CWD), maximum temperature (Tmax), and vapor pressure deficit (VPD) from the TerraClimate dataset at a 4‐km resolution (Abatzoglou et al., 2018). These were used to develop monthly *z*‐scores, standardizing the fire ignition month relative to April–June averages for a 30‐year reference period (1986–2015). This approach emphasizes site‐specific annual climatic variation rather than absolute spatial variation. Topographic variables including aspect and slope were gathered from the LANDFIRE dataset (LANDFIRE, 2020a, 2020b); aspect was circular transformed into eastness and northness prior to sampling. A vector ruggedness measure (VRM) ranging from 0 to 1, representing topographic roughness, was sampled from the USGS ScienceBase‐Catalog (Welty & Jeffries, 2018). To limit spatial autocorrelation, all weather, climate, and topographic covariates described above were sampled using 0.1% random 30‐m pixels for each day of fire spread. These values were used to create daily averages of each covariate within each DOB patch for comparison with daily spread metrics.

Because both metrics of fire spread (daily area burned and daily maximum linear spread) are log‐normally distributed, we applied a log_10_ transformation to each prior to analyses. To test for the effect of aspen cover on fire spread relative to the effects of other cover types, we developed linear mixed‐effects models as implemented by the glmmTMB package in R (Magnusson et al., 2017) with a Gaussian distribution. We first modeled the effect of aspen cover on fire spread rates using all fires with aspen presence (defined as at least 0.1% of the aspen cover class; *N* = 1687). Most fires in these models only included a very small percentage of aspen. Thus, to gain a greater understanding of the influence of aspen where it is more abundant, we next developed models using subsets of fires with greater minimum thresholds of >5% (*N* = 669) and >10% aspen cover (*N* = 404). Aspen cover was modeled as a fixed effect; fire event ID was included as a random effect to account for other differences across fires. To contrast the effect of aspen with other cover types expected to slow fire progression (e.g., prior burns), we also developed models predicting aerial and linear fire growth for each of our 10 other cover types.

To assess whether or not any effects of aspen on fire progression may be influenced by weather, climate, and topography, we also developed models predicting fire spread as a function of aspen cover that included potential interactions with these covariates. For these, a top–down model selection approach was used, beginning with an initial model including all measured covariates as first‐order terms. We then dropped all non‐significant terms to define the most influential covariates for consideration in models with second‐order interactions. A Pearson's correlation matrix was used to identify collinearity between variables, with correlated terms (*r* > 0.3) being removed such that we retained only the term that was individually more predictive. Model fit was assessed using Akaike's information criterion (AIC).

### Perimeter analysis: Relative to other vegetation types, how well does aspen stop fire?

**FIGURE 3:**
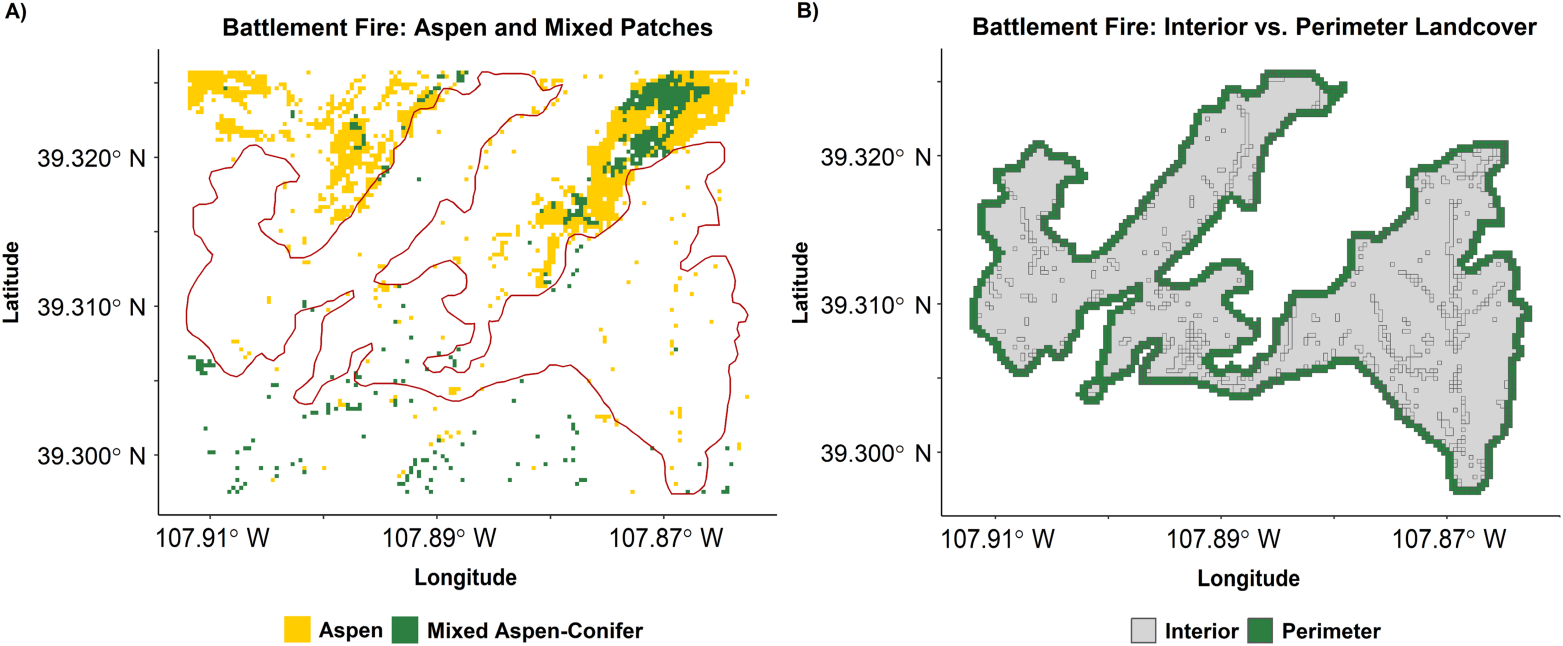
(A) Aspen cover and (B) interior versus perimeter pixels sampled for an example fire—the 2001 Battlement fire in Colorado.

To test for the effectiveness of aspen stands in stopping fire progression and determining the location of final fire perimeters, we examined the relative abundance of aspen and other vegetation types within burn interiors and at fire perimeter outer boundaries (Figure 3A) at 314 burns that occurred between 2001 (beginning with the first year of LANDFIRE EVT availability) and 2020, which included at least 0.1% of the aspen cover type. We developed perimeter/interior landcover proportions generated from MTBS fire perimeters and the most recent LANDFIRE EVT preceding the fire. Perimeter vegetation type was sampled within a 120‐m buffer (60 m on either side) of the MTBS fire perimeter (Figure 3B). This buffer distance was chosen to account for the spatial grain of the vegetation classification by allowing for up to two 30 × 30‐m landcover pixels on either side of the perimeter to be included. Consequently, this buffer captures the influence of vegetation that was burned over but may have contributed to slowing the fire before it ultimately stopped. Additionally, this buffer accounts for minor spatial error in mapped perimeters. Burn interior vegetation was sampled within the inside edge of the perimeter buffer (60‐m interior) (Figure 3B). All pixels occurring in each spatial subset (buffer or interior) were sampled to generate landcover proportions (area occupied by any given type/total area sampled) for each of the same set of 11 vegetation types described above (aspen, cool conifer, etc.).

For each of the 11 cover types, we calculated a metric of vegetation relative abundance at burn perimeter perimeters versus interiors, as follows. For any given cover type, the fire perimeter effect (FPE) is calculated:

$$\;\text{Fire Perimeter Effect}\;=\frac{\text{Perimeter Proportion}-\text{Interior Proportion}}{\text{Perimeter Proportion}+\text{Interior Proportion}}.$$

This metric is constrained between ˗1 and 1. FPE values <0 indicate a greater proportion of the cover type within the burn interior, and values >0 represent a greater proportion at the perimeter. For example, if a cover type is twice as common at perimeters compared to interiors, indicative that it impeded fire spread, it would receive an FPE of 1/3 or 0.33. By using relative rather than absolute abundance, the metric is scale independent. For each cover type, we conducted a sign test as implemented by the sign() function from the base R package, in which a negative difference (FPE below zero) or a positive difference (FPE above zero) was assigned to each vegetation type per fire; the summary of these differences (number of + and − differences across all fires) was then evaluated by an exact binomial test (R Core Team, 2023). To reduce the probability of Type I error in testing for an effect of 11 different types, we applied a Bonferroni correction to adjust our significance threshold to *p* < 0.0045 (α = 0.05/11). To assess the possible influence of fire weather and climate on aspen effects, we also tested for an effect of the daily fire weather and monthly climate variables described previously on the FPE of aspen using linear models. All weather and climate variables were sampled using 0.1% random points within each fire event, with the mean value for each used as predictor variables. Finally, we used a general additive model (GAM) as implemented in the mgcv package (Wood, 2017) to test for a nonlinear relationship between FPE and day‐of‐year (DOY). All analyses were conducted in R (R Core Team, 2023).

## RESULTS

### Aspen reduces fire spread rate

**FIGURE 4:**
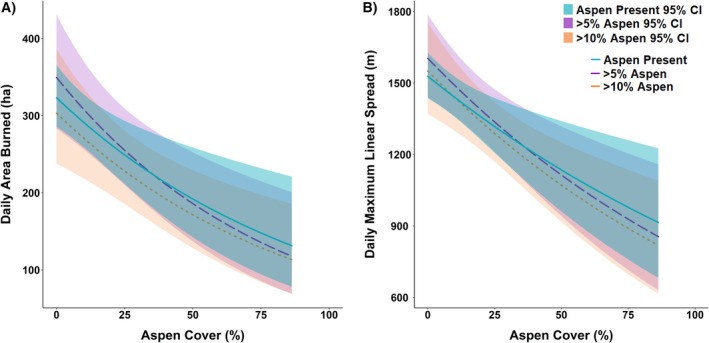
Relationships between aspen cover and (A) daily area burned (in hectares) and (B) maximum daily linear spread (in meters). Model fits represent fires with any aspen present (blue), greater than 5% aspen cover (purple), and greater than 10% aspen cover (orange). The highest aspen cover in a day‐of‐burning (DOB) patch was 86%.

Aspen cover was negatively related to both metrics of fire spread rate (Figure 4). As percent aspen cover in a DOB patch increased, both daily area burned and maximum daily linear spread decreased (*p* < 0.001; Figure 4; Appendix S1: Table S3). We found similar relationships for analyses that included the subset of fires with aspen present, aspen >5%, and aspen >10%; however, models restricted to fires with greater quantities of aspen explained more variance (higher *R* ^2^; Appendix S1: Table S3). Where aspen cover within DOB patches was <10%, mean (±1 SD) DOB patch size was 1112 ± 84 ha/day; where aspen cover was 10%–25%, daily growth dropped to 675 ± 58 ha/day; where aspen exceeded 25%, growth averaged only 368 ± 43 ha/day. Similarly, linear growth averaged 2073 ± 62 m/day where aspen cover was <10%, 1899 ± 91 m/day for aspen cover 10–25%, and 1349 ± 84 m/day for aspen cover >25%.   

Weather and climate variables were also strong and significant predictors of fire spread rates (Appendix S1: Table S3). However, no significant interactions were found, suggesting that aspen's influence on fire spread rates relative to other vegetation types was not strongly affected by weather or climate (Appendix S1: Table S4). The best‐fitting model predicting daily area burned incorporated aspen cover, FWI, and DOY (Appendix S1: Table S3). In this model, both FWI and DOY exhibited a positive relationship to daily area burned, while aspen cover was negative. The best‐fitting model predicting maximum daily linear spread incorporated aspen cover, FWI, and DOY (Appendix S1: Table S3), with positive effects of FWI and DOY but a negative effect of aspen cover.

### Aspen influences fire perimeter locations

**FIGURE 5:**
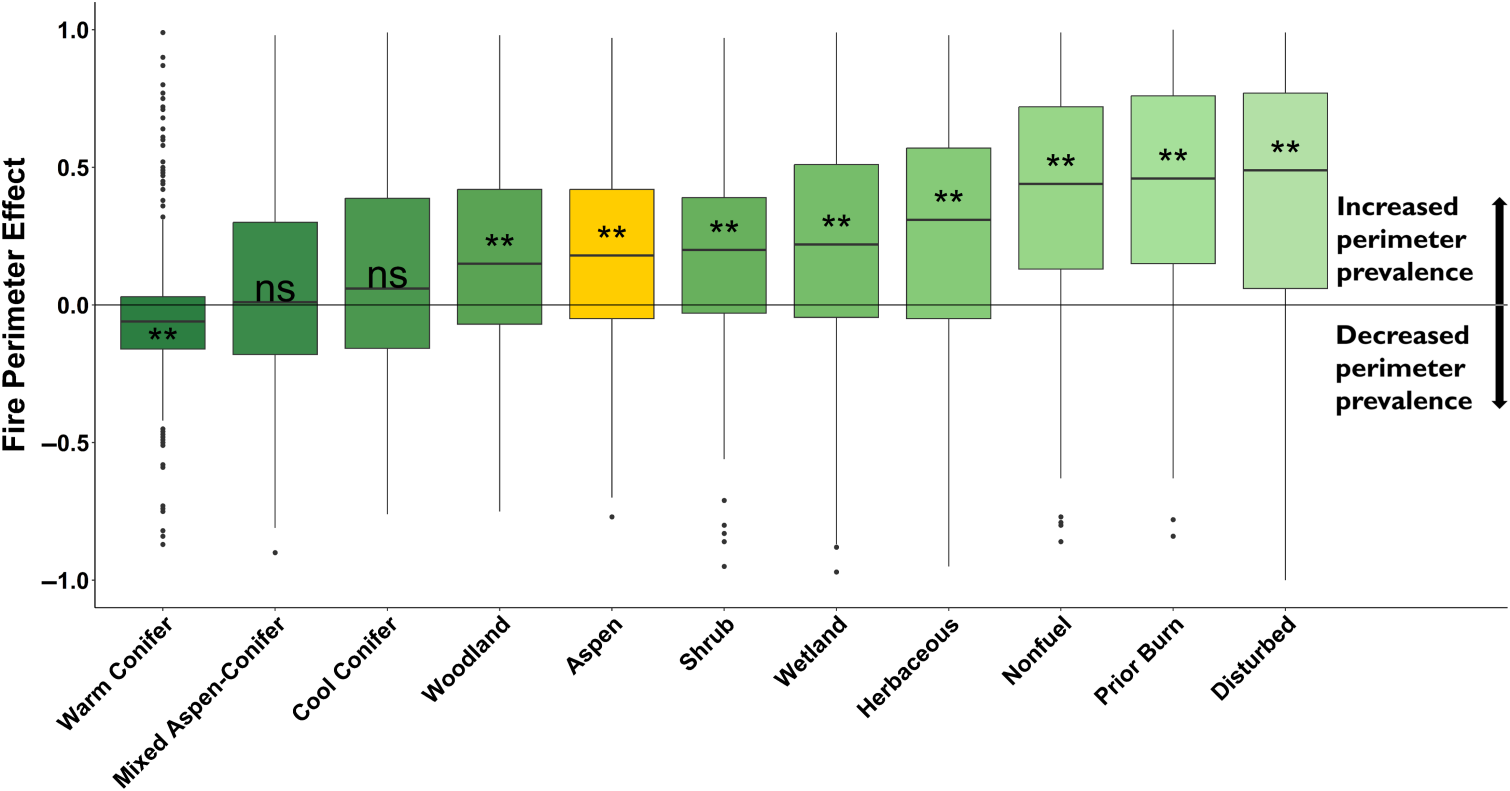
Box plot of fire perimeter effect (FPE) for each cover type across all fire events, depicting the median and distribution of FPE values (**p < 0.001; *p < 0.05; ns, *p* < 0.1).

Median values of the fire perimeter effect (FPE) across fires ranged from −0.06 for the warm conifer cover type to 0.49 for the disturbed type (Figure 5), with negative values representing increased proportion within burn interiors and positive values representing increased proportion at perimeters. The FPE of aspen was strongly positive (median FPE = 0.18; *p* < 0.001; 217 fires with positive differences and 94 with negative differences). The median aspen FPE of 0.18 is equivalent to aspen being 44% more abundant in perimeters than in burn interiors. Most other cover types had a significant positive effect (Figure 5). However, mixed aspen–conifer and cool conifer types were not significant, and the warm conifer was strongly negative (FPE = −0.06; *p* < 0.001; Figure 5).

**FIGURE 6:**
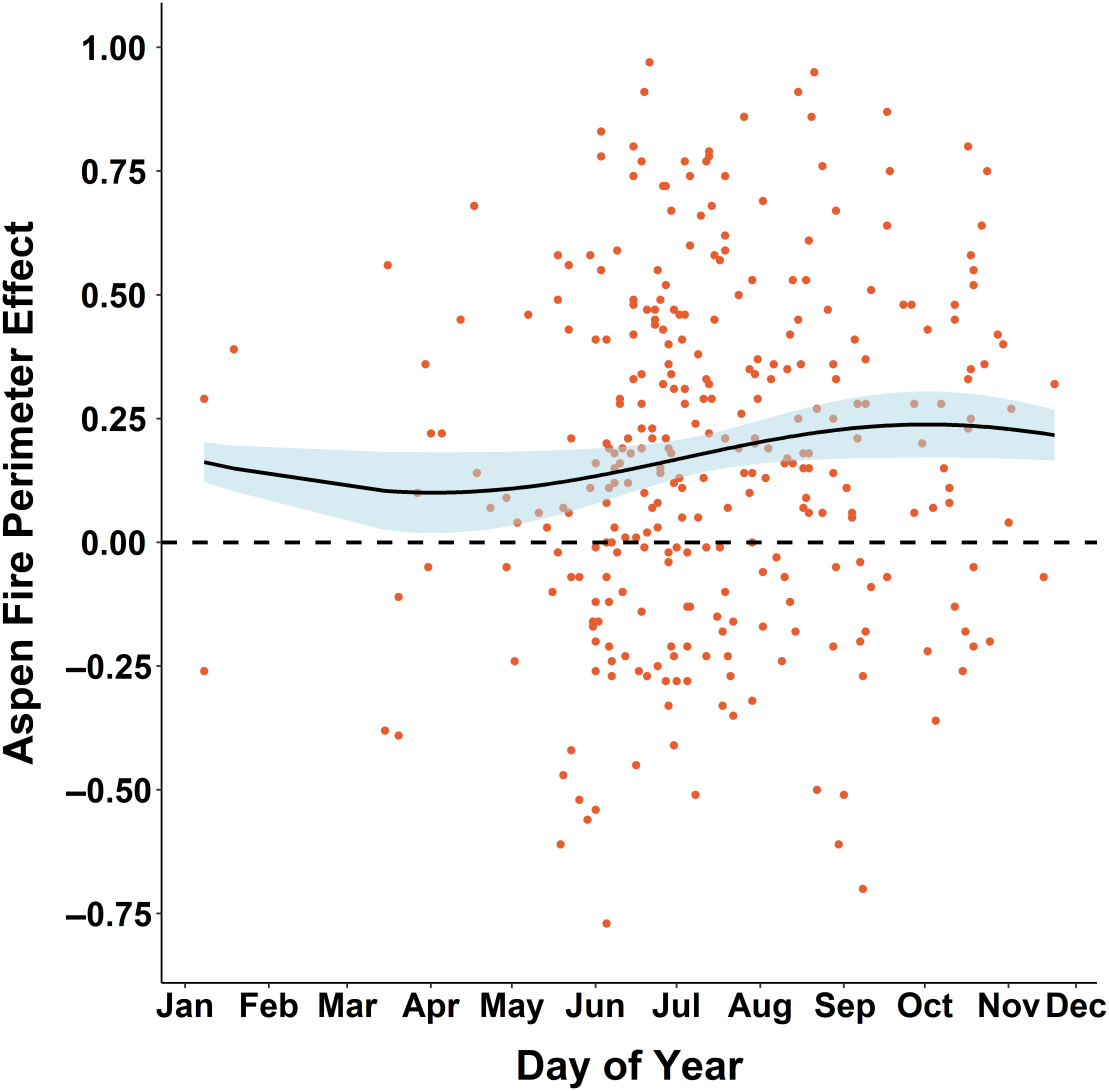
The aspen fire perimeter effect (FPE) by day‐of‐year.

The aspen FPE was not significantly predicted by any fire season climate or weather variables. However, it was weakly nonlinearly related to day‐of‐year (GAM *p* = 0.035, *r* ^2^ = 0.014, Figure 6), indicating that aspen was slightly more common on the perimeter of fires occurring in the late summer but less so for fires occurring earlier in the year.

## DISCUSSION

Aspen cover reduced fire spread rates and promoted fire perimeter formation, demonstrating how this forest type may act as a firebreak across the southwestern United States. The effect of aspen was prevalent even when wildfires were burning under the extreme weather conditions that have produced most area burned in wildfires of the past two decades. Our findings are consistent with observations (Fechner & Barrows, 1976; Nesbit et al., 2023) and expectations based on morphological differences between aspen and conifer forest types (Brown & DeByle, 1987; Shinneman et al., 2013; Tepley & Veblen, 2015). However, this study is the first to quantify these effects as they pertain to landscape‐scale patterns of fire spread and perimeter formation.

We found that increased aspen cover lowered fire spread rates, as measured by daily area burned and maximum linear spread. Lower fire spread rates in aspen forests may be due to faster decomposition of aspen leaf litter compared to conifer needles, self‐pruning canopies that are held high above the understory, and high foliar moisture in aspen leaves and understory vegetation, all of which reduce both surface and crown fire potential (Buck & St. Clair, 2012; LaMalfa & Ryle, 2008; Nesbit et al., 2023; Prescott et al., 2000). While the variation in measured fire spread rates across our study accounted for by aspen cover alone was relatively low, this is not unexpected given that a broad suite of both bottom–up (e.g., fuels and topography) and top–down factors (e.g., wind, humidity, temperature) control fire growth rates (Jolly et al., 2015; Parks et al., 2012; Potter & McEvoy, 2021). Recent studies have highlighted that, particularly under extremely warm and dry conditions, top–down controls may override bottom–up variation (Abatzoglou & Williams, 2016; Jain et al., 2022). We did not find any evidence that the effect of aspen in slowing fire spread relative to conifer forests changed under more fire‐conducive weather and drought conditions; however, additional research examining this question (e.g., by directly measuring fire spread in aspen at finer spatial scales and under varying fuel moisture and fire weather conditions) may be useful.

In addition to slowing fire growth, aspen stands appear to have stopped fire from spreading in some settings, as evidenced by the high prevalence of aspen at fire perimeters relative to burn interiors. It is also important to note that fire perimeter locations may be strongly shaped by incident management and other landscape features (e.g., natural fuel breaks, roads), which cannot be ruled out as contributing factors. However, the role of aspen in perimeter formation is most likely a function of the same factors that can reduce fire spread rates: fuel limitations and high moisture content, as described previously. This effect is expected to be greatest where aspen stands act as a barrier to crown fire spread from neighboring conifer stands (Alexander & Lanoville, 2004; Nesbit et al., 2023). Other research has demonstrated how shifts in vegetation can produce patterns of unburned islands within fire perimeters (Meddens et al., 2018), and fuel limitations can define fire perimeter formation (Holsinger et al., 2016). Here, the effect of aspen at fire perimeters was comparable to that of woodland, shrub, and wetland vegetation types, which also have much lower abundances of receptive fuels than conifer forests, but not as strong as that of our nonfuel, prior burn, and disturbed landcover types.

Although a number of fire season climate and weather factors (e.g., FWI) were strongly related to fire spread rate, we did not find any evidence that these modulated the relative effect of aspen. Instead, our analyses suggest that under more fire‐conducive conditions, fire spread rates increased in all cover types. The fires used in our analysis include many fires that burned under extreme conditions, exhibiting remarkably large runs (e.g., the 2020 East Troublesome fire). However, the coarse resolution (4‐ and 31‐km) grids of climate and weather factors may limit our ability to discern interactions with vegetation type. Our analysis suggests that the strength of aspen in slowing fire and as a perimeter‐forming fuel break may be tied to phenology, as measured by day‐of‐year, though more research on this topic could help better define the extent to which phenology modulates the flammability of aspen. Phenological processes have been shown to influence aspen flammability in the aspen parklands and boreal forests of Canada, with a period after snow melt and prior to leaf flush being more conducive to fire ignition and spread, followed by decreasing flammability after leaf‐out (Alexander, 2010; Parisien et al., 2023). In our study, the FPE of aspen appears lowest during the spring months, during which time understories may allow fire spread prior to the increasing foliar moisture associated with leaf out that then impedes spread during summer (Pickell et al., 2017). We note that the effect of aspen on perimeter locations was variable and in fact not positive for all fires. This may be due to the sensitivity of our FPE metric in cases where aspen abundance is particularly low or where the Landfire EVT maps are inaccurate, but also highlights that fire growth and cessation is subject to a wide range of influences including fuels, terrain, weather, and management (e.g., Holsinger et al., 2016) that could potentially override the aspen effect examined here.

Although we found influences of aspen cover on fire spread rates, we note that the relatively coarse vegetation classification data we relied on undoubtedly mask considerable variation in aspen stand characteristics and attendant effects on fire. In particular, aspen stand flammability may vary along successional gradients and whether the stand is seral with advancing conifer succession or stable with a more homogenous aspen composition (Rogers et al., 2014). Seral aspen stands occur throughout boreal and montane forests in the United States and are widely understood to be fire dependent, with fires killing encroaching conifers and promoting aspen vegetative regeneration; in these stands, fire probability and severity increase with conifer encroachment (Shinneman et al., 2013). By contrast, stable aspen stands with limited conifer encroachment are thought to be more rarely impacted by fire, though when these stands are situated in a flammable matrix, they can burn with patchy mixed severity (Shinneman et al., 2013). We found no influence of our mixed aspen–conifer forest type, which is likely more representative of later successional stands, in slowing fire growth and no effect of this type on fire perimeter formation.

Across portions of its range, aspen is likely to be favored by increasing fire activity and compounding disturbances, as aspen can rapidly colonize burned areas via clonal suckering, long‐range dispersal, and propagation from seed on exposed mineral soil (Andrus et al., 2021; Krasnow & Stephens, 2015; Kreider & Yocom, 2021; Wan et al., 2014). Our findings suggest that potential increases in aspen cover in high‐elevation and high‐latitude forests (Andrus et al., 2021; Kulakowski et al., 2013; Nigro et al., 2022) could produce a negative feedback that dampens increases in fire activity expected with a warmer and drier climate (Tepley et al., 2018; Zhao et al., 2024). However, land use legacies and fire suppression over the last century have reduced aspen dominance and favored conifer forest types across much of western North America (DeByle et al., 1987; Hessburg et al., 2019). Efforts to increase the abundance of aspen in these settings through prescribed fire and managed wildfires, and/or supplemental planting, could restore species habitats and key ecosystem processes. Aspen stands are often biodiversity hotspots, with increased soil moisture, organic matter, and nutrients leading to high‐quality wildlife habitat for some species (Buck & St. Clair, 2012; Oaten & Larsen, 2008; Rogers et al., 2020). Aspen stands may also provide the additional benefits of increased water yield, higher albedo, and esthetic values (Assal & Keables, 2020; LaMalfa & Ryle, 2008; Wang, 2005).

Our findings support the development and use of aspen in green fuel breaks, though additional work assessing their costs, benefits, and effectiveness relative to traditional fuel treatments will be informative. The use of aspen as a fuel break would be expected to be most effective at the landscape scale and positively related to patch size, compositional homogeneity, and stand health. The use of such treatments is inherently limited to locations within the bioclimatic range of aspen. However, similar approaches to reducing undesired wildfire activity through the addition or expansion of other fire‐resistant broadleaf tree species may be useful across a wide range of fire‐prone ecosystems globally (e.g., Oliveira et al., 2023; Wang et al., 2021), presenting opportunities for nature‐based solutions that benefit forests and human communities in an increasingly fire‐prone future.

## CONFLICT OF INTEREST STATEMENT

The authors declare no conflicts of interest.

## Supporting information


Appendix S1.

## Data Availability

Data and code (MntMattH, 2025) are available on Zenodo at https://doi.org/10.5281/zenodo.15708737.
