# Supplementary material for: Aspen impedes wildfire spread in southwestern United States landscapes
Source: Ecol Appl. 2025 Jul 7;35(5):e70061. doi: 10.1002/eap.70061 (PMC12231080; doi:10.1002/eap.70061)
Supplement: Supplementary file 1 — Appendix S1. [file EAP-35-e70061-s001.pdf]

Appendix S1

Journal: Ecological Applications

Title: Aspen impedes wildfire spread in southwestern United States landscapes

Authors: Matthew P. Harris, Jonathan D. Coop, Jared A. Balik, Jessika R. McFarland, Sean A. Parks, and Camille S. Stevens-Rumann

Table S1. Vegetation types assessed in this study for their relationship to fire spread rate or perimeter occurrence, grouped using Society of American Foresters (SAF) or Society for Range Management (SRM) codes pertaining to the Landfire Existing Vegetation Types.

Group	SAF and SRM Codes Pertaining to Landfire Existing Vegetation Types
**Aspen**	SAF 217: Aspen (EVT: "Rocky Mountain Aspen Forest and Woodland")
**Cool Conifer**	SAF 209: Bristlecone Pine, SAF 208: Whitebark Pine, SAF 219: Limber Pine, SAF 218: Lodgepole Pine, SAF 206: Engelmann Spruce-Subalpine Fir.
**Disturbed**	LF 20: Developed, LF 80: Agriculture, LF 66: Recently Burned - Herbaceous, LF 62: Recently Logged-Herbaceous, LF 63: Recently Logged-Shrub, LF 64: Recently Logged-Tree, LF 67: Recently Burned-Shrub, LF 68: Recently Burned-Tree, LF 98: Recently Disturbed Other-Herbaceous, LF 99: Recently Disturbed Other-Shrub, LF 100: Recently Disturbed Other-Tree
**Herbaceous**	LF 60: Transitional Herbacous Vegetation, SRM 410: Alpine Rangeland, SRM 735: Sideoats Grama-Sumac-Juniper, SRM 605: Sandsage Prairie, SRM 505: Grama-Tobosa Shrub, SRM 106: Bluegrass Scabland, SRM 314: Big Sagebrush-Bluebunch Wheatgrass, SRM 704: Blue Grama-Western Wheatgrass, SRM 713: Grama-Muhly-Threeawn, SRM 502: Grama-Galetta, SRM 311: Rough Fescue-Bluebunch Wheatgrass, SRM 312: Rough Fescue-Idaho Fescue, SRM 606: Wheatgrass-Bluestem-Needlegrass, SRM 409: Tall Forb, SRM 604: Bluestem-Grama Prairie, SRM 720: Sand Bluestem -Little Bluestem Dunes, SRM 611: Blue Grama-Buffalograss, SRM 601: Bluestem Prairie, LF 54: Introduced Upland Vegetation - Herbaceous, SRM 701: Alkali Sacaton-Tobosa Grass, LF 56: Western Herbaceous Wetland, LF 54: Introduced Upland Vegetation-Herbaceous
**Mixed Aspen-Conifer**	SAF 217: Aspen (EVT: "Inter-Mountain Basins Aspen-Mixed Conifer Forest and Woodland")
**Nonfuel**	LF 11: Water, LF 12: Snow-Ice, LF 31: Barren, LF 33: Sparsely Vegetated, Non-vegetated, No Dominant Lifeform
**Prior Burn**	Any landcover that burned within the prior 10 years (within MTBS fire perimeters)
**Shrub**	SAF 241: Western Live Oak, SRM 415: Curlleaf Mountain-Mahogany, SRM 405: Black Sagebrush, SRM 501: Saltbush-Greasewood, SRM 506: Creosotebush-Bursage, SRM 414: Salt Desert Shrub, SRM 729: Mesquite, SRM 212: Blackbush, SRM 406: Low Sagebrush, SRM 403: Wyoming Big Sagebrush, SRM 421: Chokecherry-Serviceberry-Rose, SRM 508: Creosotebush-Tarbush, SRM 503: Arizona Chaparral. SRM 413: Gambel Oak, SRM 507: Palo Verde-Cactus, SRM 402: Mountain Big Sagebrush, LF 41: Deciduous Shrubland, SRM 730: Sand Shinnery Oak, LF 52: Introduced Upland Vegetation-Shrub
**Warm Conifer**	SAF 213: Grand Fir, SAF 210: Interior Douglas-Fir, SAF 211: White Fir, SAF 237: Interior Ponderosa Pine, SAF 238: Western Juniper

**Wetland**	SAF 235: Cottonwood-Willow, SRM 203: Riparian Woodland, SRM 422: Riparian, LF 42: Great Plains Riparian, LF 58: Introduced Woody Wetlands and Riparian Vegetation, LF 59: Introduced Herbaceous Wetland and Riparian Vegetation
**Woodland**	SRM 418: Bigtooth Maple, SRM 504: Juniper-Pinyon Pine Woodland, SRM 412: Juniper-Pinyon Woodland

Table S2. Number and percentage of landcover pixels sampled in the perimeter analysis, by landcover group, including both perimeter and interior pixels.

Landcover Group	Number of pixels sampled	Percent of pixels sampled
**Aspen**	1,145,068	5.9%
**Cool Conifer**	3,310,109	17%
**Disturbed**	361,876	1.9%
**Herbaceous**	1,162,013	6%
**Mixed Aspen-Conifer**	1,373,651	7.1%
**Nonfuel**	233,543	1.2%
**Prior Burn**	512,817	2.7%
**Shrub**	1,911,227	10%
**Warm Conifer**	7,032,904	36.4%
**Wetland**	347,932	1.8%
**Woodland**	1,936,403	10%

Table S3. Linear models for fires with aspen present, >5% aspen, >10% aspen, and the top model of aspen present plus covariates, for both metrics of fire spread. The top model was identified by dropping non-significant terms and minimizing AIC using a top-down selection approach. Marginal R^2 denotes variation explained by fixed effects and the conditional R^2 includes variation explained by the fixed effects and random effect of event ID. * Models with an NA* cannot be compared using AIC due to having a different sample size. Sin.doy refers to the day of the year which was circular transformed using sin().

Model	AIC	Marginal R^2	Conditional R^2	N
a) Daily Area Burned				
~ % Aspen + (1|Event_ID)	2704	0.01	0.26	1687
~ >5% Aspen + (1|Event_ID)	NA*	0.02	0.26	669

~ >10% Aspen + (1|Event_ID)	NA*	0.03	0.27	404
~ % Aspen + fwi + sin.doy + (1|Event_ID)	2510	0.18	0.41	1687
b) Maximum Daily Linear Spread				
~ % Aspen + (1|Event_ID)	914.6	0.01	0.16	1687
~ >5% Aspen + (1|Event_ID)	NA*	0.02	0.17	669
~ >10% Aspen + (1|Event_ID)	NA*	0.03	0.13	404
~ % Aspen + fwi + sin.doy + (1|Event_ID)	791.5	0.13	0.28	1687

Table S4. Model selection table depicting the percent aspen only model and the top 5 best fit models incorporating the addition of or interaction with individual terms for all spread variables. Metrics with ^{Int} in front of them refer to an interaction effect.

**Model**	**AIC**	r^2	β_0 Intercept	β_1 Slope Eff. 1	β_2 Slope Eff. 2 or *Interaction Eff.	*P*-Value
**Daily Area Burned**						
**~ % Aspen**	2704	0.01	323 ha	-0.005	NA	0.0013
**~ % Aspen + FWI**	2521.3	0.145	95 ha	-0.005	0.011	P<0.001
**~ % Aspen * FWI**	2521.7	0.147	90 ha	-0.001	0.012	^{Int} NS *(P*=0.2)
**~ % Aspen + ISI**	2522.8	0.126	136 ha	-0.004	0.021	0.00103
**~ % Aspen + DSR**	2529	0.14	163 ha	-0.005	0.011	P<0.001
**~ % Aspen * DSR**	2530	0.141	159 ha	-0.003	^{Int} -0.00008	^{Int} NS (*P*=0.293)
**Maximum Daily Linear Spread**						
**~ % Aspen**	914.6	0.01	1529 m	-0.003	NA	0.0013

**~ % Aspen + ISI**	787.9	0.01	1003 m	-0.003	0.011	0.0014
**~ % Aspen + DSR**	803.6	0.01	1115 m	-0.003	0.005	0.00101
**~ % Aspen *DSR**	805.1	0.01	1104 m	-0.002	[Int] -0.000033	[Int] NS (*P*=0.48)
**~ % Aspen + FWI**	805.5	0.01	885 m	-0.003	0.005	P<0.001
**~ % Aspen * FWI**	806.3	0.01	861 m	-0.0005	[Int] -0.000047	[Int] NS (*P*=0.27)
